# Rhein promotes TRAIL-induced apoptosis in bladder cancer cells by up-regulating DR5 expression

**DOI:** 10.18632/aging.204236

**Published:** 2022-08-19

**Authors:** Liang Ma, Hong-Ling Wei, Ke-Jie Wang, Xiang-Yu Meng, Sai-Qi Ni, Cheng Zhou, Yi Li, Rui Yu, Qi Ma

**Affiliations:** 1 Medical School, Ningbo University, Ningbo, Zhejiang 315211, China; 2 Comprehensive Urogenital Cancer Center, Ningbo First Hospital, The Affiliated Hospital of Ningbo University, Ningbo, Zhejiang 315010, China; 3 Translational Research Laboratory for Urology, The Key Laboratory of Ningbo City, Ningbo First Hospital, The Affiliated Hospital of Ningbo University, Ningbo, Zhejiang 315010, China; 4 Ningbo Clinical Research Center for Urological Disease, Ningbo, Zhejiang 315010, China; 5 Department of Urology, Ningbo First Hospital, The Affiliated Hospital of Ningbo University, Ningbo, Zhejiang 315010, China; 6 Department of Urology, The Second Affiliated Hospital, School of Medicine Zhejiang University, Hangzhou, Zhejiang 310009, China

**Keywords:** bladder cancer, drug resistance, Rhein, TRAIL, DR5

## Abstract

Tumor necrosis factor-related apoptosis-inducing ligand (TRAIL) combined with sensitizer is a potential method to reverse TRAIL-resistance in tumor cells. Rhein (RH) is a monomer extracted from Chinese herbs that has been reported to show anti-tumor effects in a variety of tumor cells, but the role of RH in TRAIL-induced anti-tumor effects in bladder cancer cells has not been reported. In this study, we found that the combined treatment of a non-toxic concentration of RH with TRAIL significantly inhibited the proliferation and induced apoptosis in both TRAIL sensitive and resistant bladder cancer cell lines. Furthermore, we found that RH promoted bladder cancer cell apoptosis by up-regulating DR5 expression. Our findings provide potential value in the clinical treatment of bladder cancer.

## INTRODUCTION

Bladder cancer is one of the most common malignant tumors found in the urological system [1, 2]. 70–80% of patients are found to have non-muscle invasive bladder cancer (NMIBC) when they are diagnosed. The treatment of NMIBC usually requires transurethral resection of bladder tumor (TURBT) and postoperative adjuvant intravesical chemotherapy. For patients with a high risk of recurrence, Bacillus Calmette-Guerin (BCG) Vaccine intravesical infusion is also recommended. Postoperative bladder infusion with BCG can reduce the recurrence rate of NMIBC [3], but the side effects of BCG such as bladder irritation, allergy, sepsis, and even shock have limited its use in clinical practice. Ludwig et al. [4] found soluble tumor necrosis factor-related apoptosis-inducing ligand (TRAIL) in the urine of NMIBC patients who received intravesical infusion of BCG, and the expression of TRAIL also significantly increased in patients who had a good response to BCG. Therefore, TRAIL may be an important effector in the BCG treatment for NMIBC patients.

TRAIL, as a member of tumor necrosis factor (TNF) superfamily, was amplified from the atrial myocyte cDNA library by Wiley [5] in 1995. TRAIL is widely expressed in various cells of the immune system, and has been reported to inhibit tumorigenesis through natural killer (NK) cells, T cells, etc [6]. Several studies have shown that TRAIL selectively and rapidly induce tumor cell apoptosis, without causing significant cytotoxicity in normal cells [7, 8], which makes it a potential anti-tumor agent. Gazitt [9] also reported that TRAIL selectively killed multiple myeloma cells, but had no significant effect on hematopoietic stem cells. Although TRAIL has several advantages, it has limited clinical applications due to intrinsic resistance to TRAIL-induced apoptosis in cancer cells. Griffith et al. [10] found five human melanoma cell lines (WM 9, WM 35, WM 98-1, WM 793, and WM 1205 Ln) were sensitive to TRAIL, while the other three cell lines (WM 164, WM 1791-C, and WM 3211) were resistant to TRAIL. Another study also found that five colorectal cell lines (SW480, HCT116, KM12C, Caco-2, and DLD-1) were resistant to TRAIL [11]. Szliszka et al. [12] found that bladder cancer cell line SW780 was sensitive to TRAIL, while 647-V and T24 were resistant to TRAIL. Although many malignant tumors are resistant to TRAIL, some studies have shown that when TRAIL was used in combination with Chinese herbal monomers, small molecule inhibitors, or chemotherapeutic agents, TRAIL resistance can be reversed. Therefore, the combination of TRAIL with an effective sensitizer may be a feasible strategy for the treatment of TRAIL-resistance bladder cancer.

**Figure 1:**
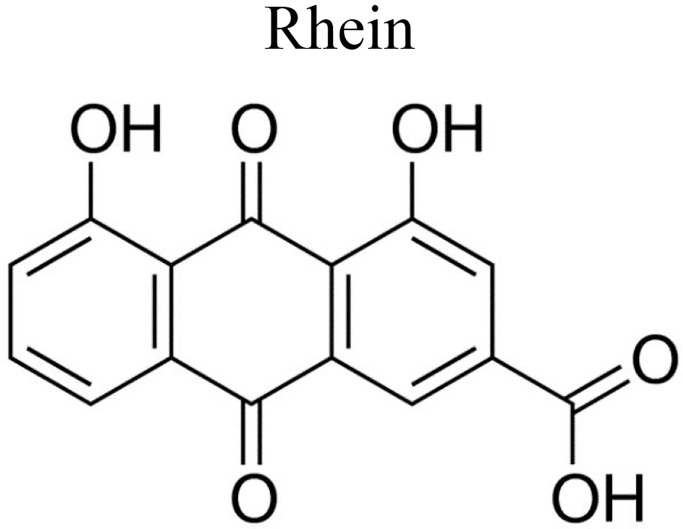
**The molecular structure of RH.** Rhein, also called 1,8-dihydroxy-3-carboxy anthraquinone, is abbreviated as RH.

Medicinal herbs have several obvious advantages in combination treatment, including enhancing the therapeutic effects and attenuating side effects [13, 14]. Due to the advantages given by herbs, we preferred to use herbs as the sensitizers for TRAIL treatment. Rhein (RH, C15H8O6, MW 284.22) (Figure 1) is a monomer extracted from traditional Chinese herbs, such as *Polygonum cuspidatum*, *Polygonum multiflorum* or *Rheum rhabarbarum*. Previous studies showed that RH exhibited anti-tumor effects by inducing apoptosis in multiple tumor cells [15–20]. In our study, we found that a combination treatment of RH with TRAIL significantly inhibited the proliferation and induced apoptosis in bladder cancer cell lines that were intrinsically resistant to TRAIL. Furthermore, RH promoted cell apoptosis of TRAIL-resistant bladder cancer cells via the up-regulation of DR5 expression. These findings indicate that RH plus TRAIL is a potential treatment strategy for human bladder cancer.

## MATERIALS AND METHODS

### Reagents

RH (purity ≥98%) was obtained from Sigma-Aldrich (St. Louis, MO, USA). Recombinant human TRAIL was obtained from Peprotech (Rocky Hill, NJ, USA). Cell Counting Kit-8 (CCK-8) assay was purchased from Dojindo Molecular Technologies, Inc. (Dojindo Laboratories, Inc., Kumamoto, Japan). Antibodies against DR5, DR4, caspase-8, and caspase-3 were purchased from Cell Signaling Technology (Danvers, MA, USA). Glyceraldehyde 3-phosphate dehydrogenase (GAPDH) was purchased from Abcam (Cambridge, MA, USA). Phycoerythrin (PE)-conjugated antibodies against DR4, DR5, and the human IgG isotype control were from R&D Systems (Minneapolis, MN, USA). The Annexin V-FITC/PI apoptosis kit was purchased from Multi Sciences (Lianke) Biotechnology (Hang Zhou, Zhejiang, China). Fetal bovine serum (FBS), RPMI-1640, DMEM, and Lipofectamine 2000 were purchased from Thermo Fisher Scientific, Inc. (Waltham, MA, USA).

### Cell lines and culture

The human 5637 bladder cell line was obtained from the Type Culture Collection of the Chinese Academy of Sciences (Shanghai, China). Human bladder cancer cell line T24, BIU 87, and human bladder epithelial immortalized cell line SV-HUC-1 were purchased from American type culture collection, (Manassas, VA, USA). All cells were cultured at 37°C in a humidified atmosphere containing 5% CO_2_ in RPMI-1640 supplemented with 10% FBS, 2 mM L-glutamine, and 1% penicillin/streptomycin.

### Cell viability assay

The CCK8 assay was used to measure cell viability of cells treated with RH, TRAIL, or their combination. 1 × 10^4^ cells/well were seeded in 96-well plates in 100 μL medium per well, and cultured for 24 h. After drug treatments, the cells were incubated with 10% CCK8 solution in 100 μL medium for another 2 h at 37°C. The absorbance was measured at 450 nm using a SpectraMax M5 (Molecular Devices, San Jose, CA, USA).

### RNA extraction and quantitative real time-PCR (qRT-PCR)

Total RNA was extracted from at least 5 × 10^6^ cells using TRIzol reagent (Invitrogen, Karlsruhe, Germany). Then, total RNA was quantified using a NanoDrop ND2000 (Thermo Fisher Scientific, Wilmington, DE). After quantification, cDNA was synthesized by reverse transcription (RT) using random primers and the ReverTra Ace qPCR RT Master Mix with gDNA Remover (Toyobo, Osaka, Japan). The real-time PCR was conducted using the GoTaq qPCR Master Mix (Promega, Madison, WI, USA) on an Mx3005P real-time PCR System (Stratagene, La Jolla, CA, USA). The sequence of primers used for real-time PCR is listed in Supplementary Table 1.

### Flow cytometry

The rate of apoptosis was measured by Annexin V/PI assay and analyzed by flow cytometer (FACSCalibur, BD Biosciences). For measurement of receptor expression, 1 × 10^6^ cells were re-suspended in PBS, then incubated with PE-conjugated antibodies for 30 minutes at room temperature. PE-conjugated human IgGs were used for control. Finally, the flow cytometer (FACSCalibur, BD Biosciences) was used to evaluate the fluorescence intensities of the cells, and the FlowJo software (FlowJo LLC, OR, USA) was used to analyze these data.

### Western blot

The cells were washed with PBS, and then lysed with RIPA buffer (Solarbio, Beijing, China). The lysates were centrifuged at 12,000 rpm for 30 min at 4°C. Subsequently, the supernatant fractions were collected. The Bradford assay was used to measure protein concentration. An equal amount of protein was loaded and separated on 12% SDS-PAGE gels and transferred onto PVDF membranes (Millipore, Danvers, MA, USA). The membranes were incubated in primary antibodies (diluted according to manufacturer’s instructions) and then the corresponding secondary antibodies. An enhanced chemiluminescence (ECL) kit (Thermo Scientific, Rockford, CA, USA) was used to visualize the protein bands, and the Quantity One software (Bio-Rad, Berkeley, CA, USA) was used to quantify protein by densitometry.

### RNA interference

DR5 small interfering RNAs (siRNAs) and a scrambled siRNA were synthesized by GenePharma Technologies (Shanghai, China) and transfected into cells using Lipofectamine 2000.

### Statistical analysis

PASW Statistics software (version 18; SPSS Inc., Chicago, IL, USA) was used for statistical analysis. The data were expressed as the means ± standard error. One-way ANOVA was performed to examine the differences between the groups. The statistical difference was considered significant at *P* < 0.05.

### Data availability statement

All the data used to support the findings of this study are included within the article and supplement materials.

## RESULTS

### Different sensitivity of TRAIL in bladder cancer cell lines

**Figure 2:**
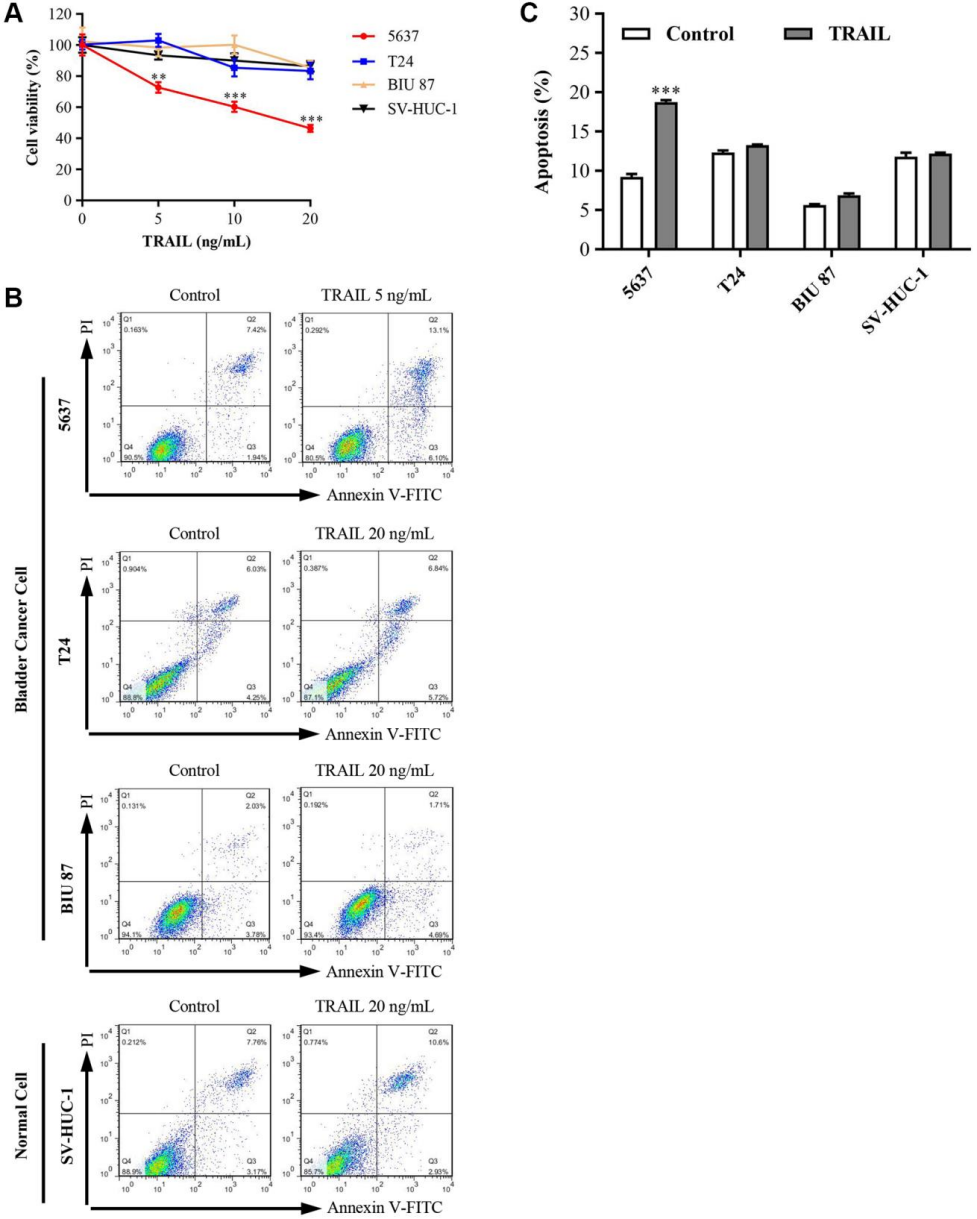
**Effects of TRAIL on cell viability and apoptosis in 5637, T24, BIU 87, and SV-HUC-1 cells.** (**A**) Bladder epithelial cell (SV-HUC-1) and bladder cancer cell lines (5637, T24 and BIU 87) were treated with different concentrations of TRAIL (0, 5, 10 and 20 ng/mL) for 24 h, and cell viabilities were detected by CCK8 kit. (**B** and **C**) According to the sensitivity of bladder cells to TRAIL, 5637 cells were treated with 5 ng/mL TRAIL, T24, BIU 87 and SV-HUC-1 cells were treated with 20 ng/mL TRAIL. After 24 h of treatment, apoptosis was detected using the Annexin V-FITC/PI apoptosis kit. The ANOVA followed by multiple pairwise comparisons using a one-way Student’s *t*-test procedure was used to analyze differences between treatment and control groups. The results represent the means ± SD of three replicates, ^**^P < 0.01, ^***^P < 0.001.

To determine the effects of TRAIL on the viability of bladder cancer cells, the CCK8 was used to detect cell viabilities. Bladder normal epithelial cell (SV-HUC-1) and bladder cancer cell lines (5637, T24, and BIU 87) were treated with different concentrations of TRAIL (0, 5, 10, and 20 ng/mL) for 24 h and then cell viabilities were measured. The results showed that 5 ng/mL of TRAIL significantly inhibited the proliferation of 5637 cells (72.67 ± 6.03); however, 5 ng/mL of TRAIL did not affect cell proliferation in T24 cells (103.00 ± 4.36), BIU 87 cells (98.36 ± 7.84) and SV-HUC-1 cells (88.00 ± 2.65) (Figure 2A). Subsequently, we investigated the effect of TRAIL on cell apoptosis. The results showed that apoptotic cells significantly increased in 5637 cells after treatment with 5 ng/mL TRAIL for 24 h. Meanwhile, no significant change was observed in apoptosis of bladder cancer cells (T24 and BIU 87) or bladder epithelial cells SV-HUC-1 (Figure 2B and 2C). Even when treated with higher concentrations of TRAIL (20 ng/mL), cell viability and apoptosis did not change significantly (Figure 2A–2C). Based on these results, T24, BIU 87, and SV-HUC-1 cells were regarded as intrinsic resistant to TRAIL.

### 10 μg/mL of RH was non-toxic concentration for normal bladder epithelial cells

**Figure 3:**
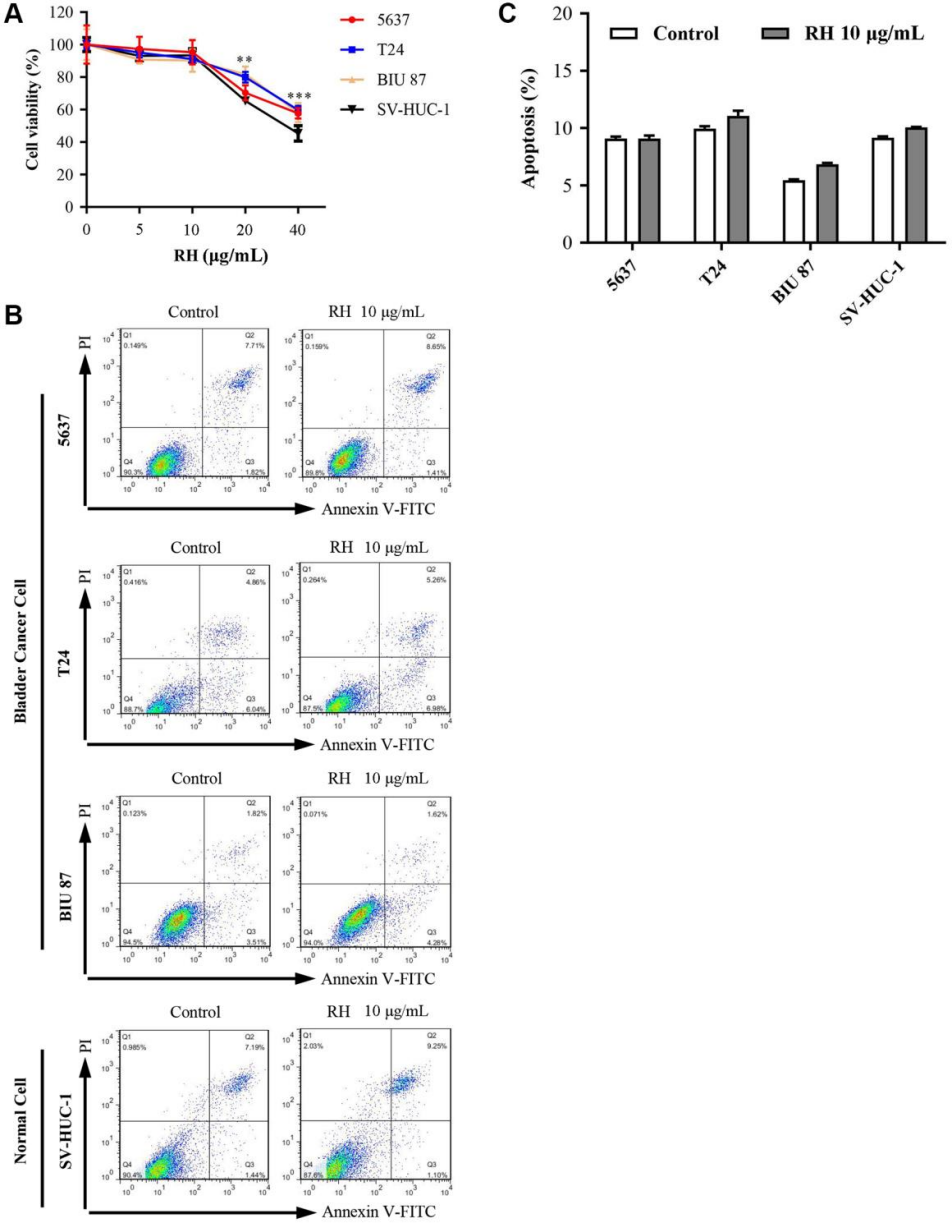
**Cell viability and apoptosis in 5637, T24, BIU 87, and SV-HUC-1 cells by treated with RH.** (**A**) After being treated with different concentrations of RH (0, 5, 10, 20 and 40 μg/mL) for 24 h, cell viability of 5637, T24, BIU 87 and SV-HUC-1 cells was measured with CCK8. (**B** and **C**) Treated with RH (10 μg/mL) for 24 h, cell apoptosis of 5637, T24, BIU 87 and SV-HUC-1 were detected using FCM. The ANOVA followed by multiple pairwise comparisons using a one-way Student’s *t*-test procedure was used to analyze differences between treatment and control groups. The results represent the means ± SD of three replicates, ^*^P < 0.05, ^**^P < 0.01, ^***^P < 0.001.

We detected the cytotoxicity of RH to SV-HUC-1 and bladder cancer cells. Cells were treated with different concentrations of RH (0, 5, 10, 20, and 40 μg/mL) for 24 h. CCK8 was then used to test cell viabilities. The results showed that as the concentration of RH increased, all cell lines were inhibited significantly. RH at 20 μg/mL significantly inhibited the proliferation of 5637 (70.33 ± 4.62), BIU-87 (81.73 ± 4.71), T24 (79.95 ± 3.32), and SV-HUC-1 (65.33 ± 2.52). However, cell viabilities in all cell lines were not affected when the concentration was under 10 μg/mL of RH (Figure 3A). These results indicated that 10 μg/mL is a cytotoxicity threshold of RH, for concentration below 10 μg/mL showed little cytotoxicity to any of the four cell lines tested (Figure 3A). We also verified that 10 μg/mL of RH was not cytotoxic to all four cell lines by detecting the apoptosis using Flow cytometry (FCM) (Figure 3B and 3C). Therefore, we believed that 10 μg/mL of RH is non-toxic concentration for bladder cancer cells and normal epithelial cells. And, 10 μg/mL of RH was used for subsequent experiments.

### RH enhanced the sensitivity of TRAIL in TRAIL-resistant bladder cancer cells

**Figure 4:**
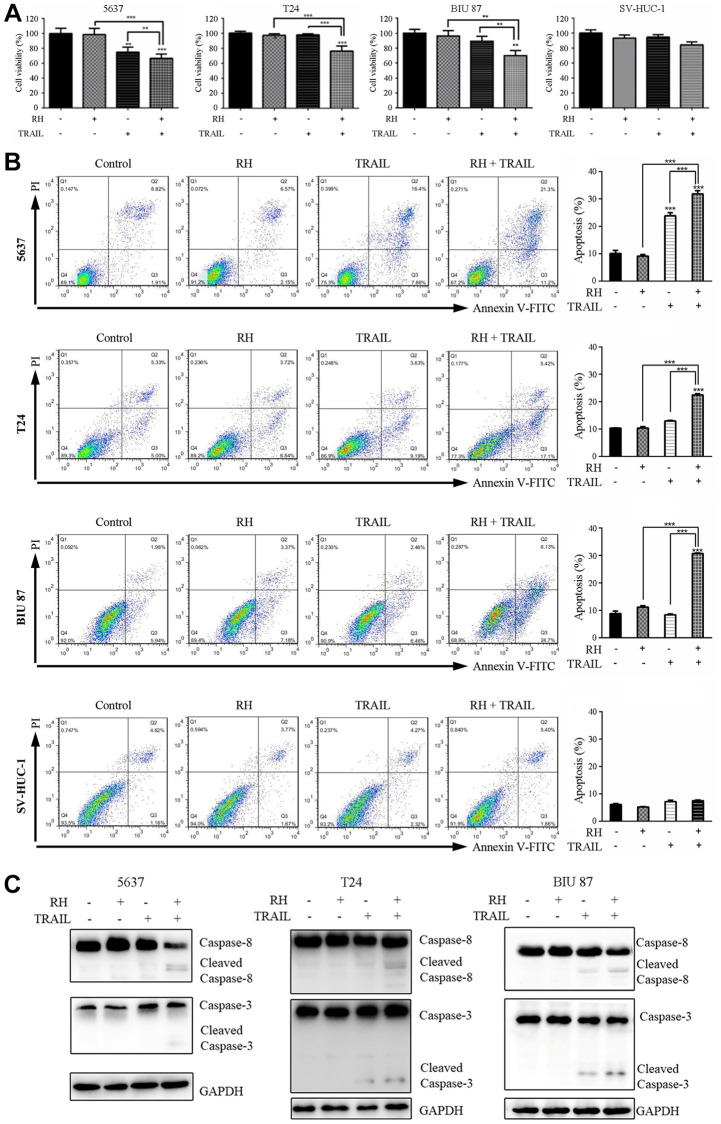
**Cell viability, apoptosis, and caspase changes in 5637, T24, BIU 87, and SV-HUC-1 cells by treatment with non-toxic RH and TRAIL.** Four bladder cell lines were treated with non-toxic RH (10 μg/mL) and different concentrations of TRAIL, TRAIL-sensitive cells (5637 cells, 5 ng/mL), and TRAIL-resistant cells (T24, BIU 87 and SV-HUC-1, 20 ng/mL) for 24 h. (**A**) Cell viabilities were measured by CCK8. (**B**) Cell apoptosis was detected via FCM. (**C**) The activation levels of Caspase-8 and Caspase-3 were detected via Western blot. Differences between groups were examined using one-way ANOVA. The results represent the means ± SD of three replicates, ^**^P < 0.01, ^***^P < 0.001.

To examine if non-toxic concentration of RH enhances the sensitivity of the bladder cancer cells to TRAIL-mediated apoptosis, we treated TRAIL-sensitive bladder cancer cells 5637 with 10 μg/mL of RH and 5 ng/mL TRAIL combination, and TRAIL-resistant bladder cancer cells T24, BIU 87 with 10 μg/mL of RH and 20 ng/mL TRAIL combination. Normal epithelial cells SV-HUC-1 cells were also treated with 10 μg/mL of RH and 20 ng/mL TRAIL combination as the control. We were surprised to find that the combination therapy significantly inhibited the growth of bladder cancer cells, especially in TRAIL-resistant tumor cells T24 and BIU 87, whereas this phenomenon was not observed in SV-HUC-1 (Figure 4A). Meanwhile, we found that TRAIL plus RH enhanced cell apoptosis in all three bladder cancer cell lines, but not in SV-HUC-1 (Figure 4B). Moreover, this combination strongly increased the levels of cleaved caspase-8 and cleaved caspase-3 in all three bladder cancer cells (Figure 4C). These results indicated that combined treatment significantly inhibited cell growth and enhanced TRAIL-induced apoptosis via activation of the caspase cascade in bladder cancer cells, but exhibited no sensitization effect on normal bladder epithelial cells.

### RH enhanced TRAIL-induced apoptosis in bladder cancer cells by up-regulating DR5 expression

**Figure 5:**
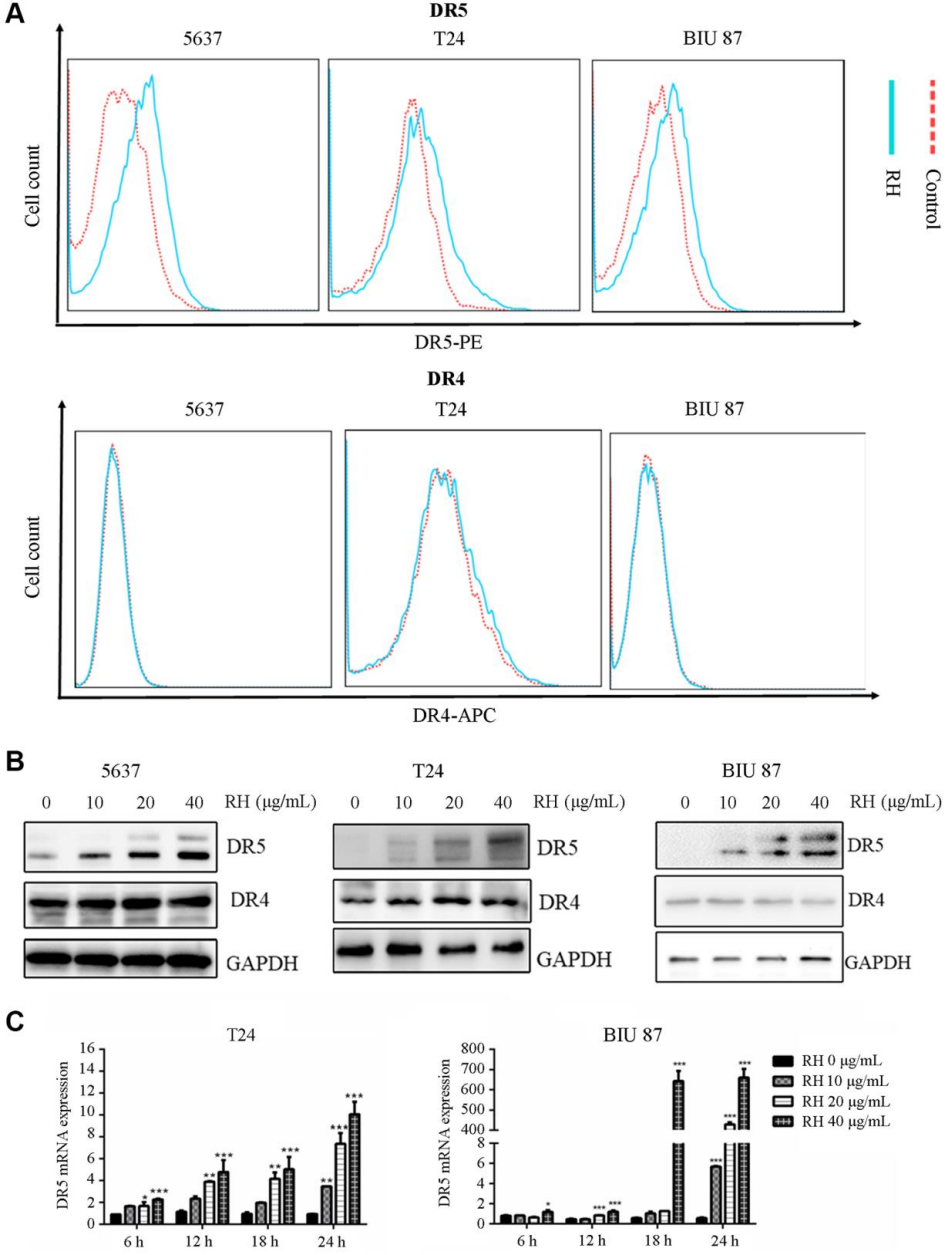
**The changes of DR5 and DR4 in bladder cancer cells by treated with RH.** (**A**) The cell surface DR5 and DR4 of T24, 5637 and BIU 87 cells were measured via FCM after treatment with RH (10 μg/mL) for 24 h. (**B**) TRAIL-sensitive cells (5637) and TRAIL-resistant cells (T24 and BIU 87) were treated with different concentrations of RH (0, 10, 20 and 40 μg/mL) for 24 h, total protein was extracted, DR4 and DR5 were detected by Western blot. (**C**) TRAIL-resistant cells (T24 and BIU 87) were harvested after being treated with different concentrations of RH (0, 10, 20 and 40 μg/mL) for 6, 12, 18 and 24 h. Then total RNA was extracted, and DR5 was detected by qRT-PCR. Differences between groups were examined using one-way ANOVA. The results represent the means ± SD of three replicates, ^*^P < 0.05, ^**^P < 0.01, ^***^P < 0.001 versus control group.

DR4 and DR5 are membrane death receptors for TRAIL-induced apoptosis [21, 22]. To further determine whether RH induces apoptosis via regulating the expression of DR4 and DR5, we detected the endogenous expression of DR4 and DR5 using FCM. First, we treated TRAIL-sensitive (5637) and TRAIL-resistant (T24 and BIU 87) bladder cancer cells with RH (10 μg/mL). After treatment, we found that the surface DR5 expression increased in all three cell lines (Figure 5A up), but no significant change in DR4 levels. (Figure 5A down). Next, we investigated the change in DR4 and DR5 protein using western blot, and the results confirmed the results from the FCM assay. RH increased the expression of DR5 protein in bladder cancer cells in a dose-dependent manner, but not for DR4 (Figure 5B). Additionally, we detected DR5 expression at transcriptional level using real-time PCR. Results showed that RH increased the level of DR5 mRNA, indicating that RH regulated DR5 expression at transcriptional level (Figure 5C).

### Silencing DR5 expression could reverse the sensitization effect of RH on TRAIL-resistant bladder cancer cells

**Figure 6:**
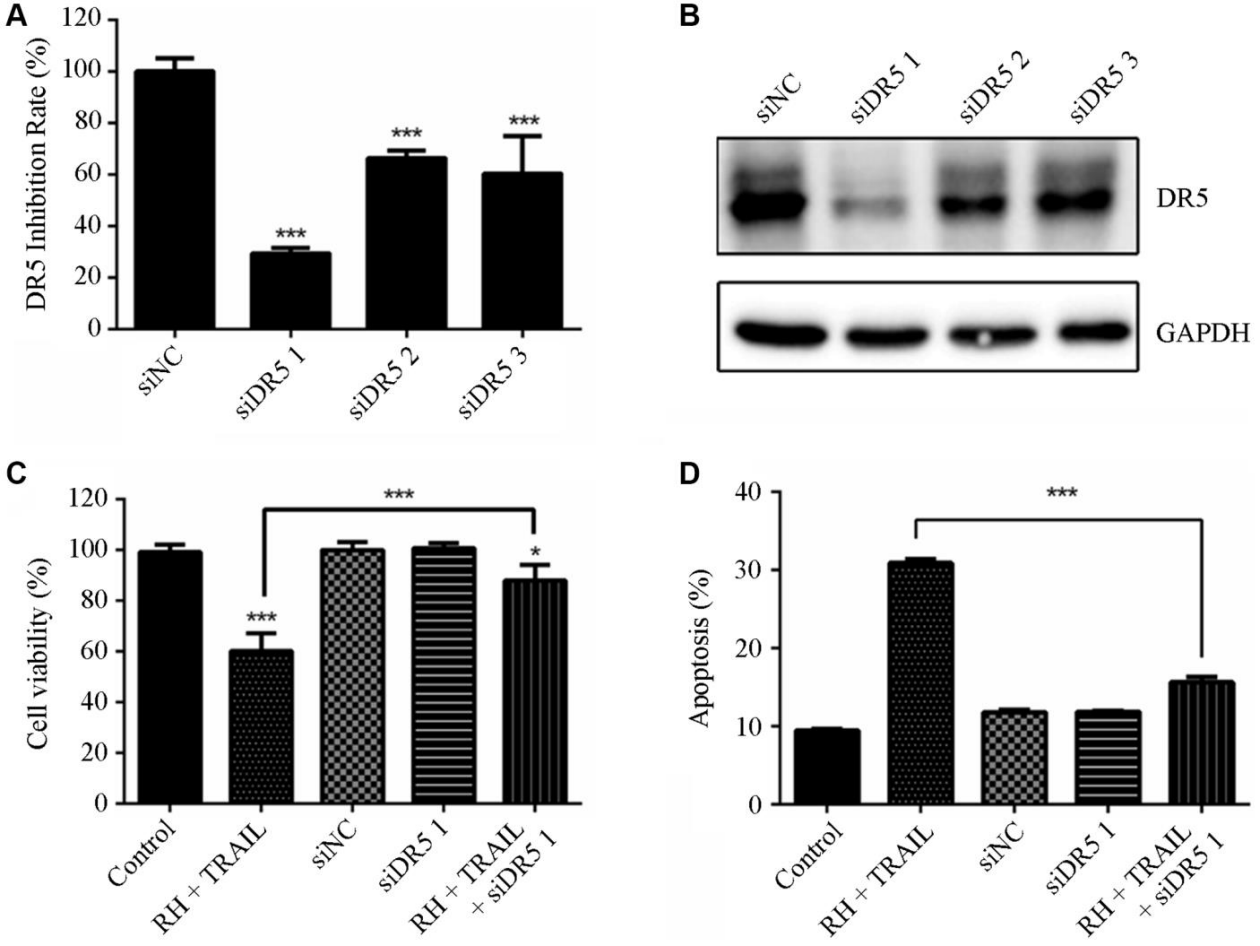
**Effect of combination treatment RH and TRAIL on cell viability and apoptosis after silencing DR5.** (**A** and **B**) Three small interfering RNA pairs were designed and verified for interference efficiency with DR5 mRNA (**A**) and protein expression in BIU 87 cells (**B**). (**C** and **D**) Transfection of TRAIL-resistant BIU 87 cells for 24 h with the siDR5 1, then RH was added. Cell viabilities were detected using CCK8 (**C**), and apoptosis was detected using Annexin V-FITC/PI (**D**). Differences between groups were examined using one-way ANOVA. The results represent the means ± SD of three replicates, ^*^P < 0.05, ^***^P < 0.001.

To validate the role of DR5 in the sensitization of TRAIL-resistant cells after treatment with RH, we designed and synthesized three small interfering RNAs targeting DR5 (named siDR5 1, siDR5 2 and siDR5 3, respectively) (Supplementary Table 2). Next, we transfected the TRAIL-resistant BIU 87 cells with the siRNAs for 24 h. Results showed that siDR5 1 significantly inhibited DR5 mRNA and protein expression (Figure 6A and 6B). Figure 6C and 6D showed that TRAIL-induced cytotoxicity and apoptosis were both inhibited in siDR5 1 transfected cells (BIU 87). Therefore, DR5 is likely to be a key molecule in RH induced sensitization of TRAIL-resistant cells.

## DISCUSSION

BCG infusion is usually used to treat high risk NMIBC, but the side effects of BCG limit its application during clinical practices. Previous studies found that TRAIL, as a potential anti-tumor agent in preclinical models, is essential in BCG-induced anti-tumor effects. However, TRAIL-resistance is the major obstacle to its application [23, 24]. Fortunately, some reports have shown that TRAIL resistance could be reversed by herbs [25–28]. RH, which is extracted from *Polygonum cuspidatum*, *Polygonum multiflorum* or *Rheum rhabarbarum*, has been found to show various pharmacological effects, including anti-tumor effects in a variety of tumor cells. However, its role in reversing the sensitivity of TRAIL-resistant bladder cells remains obscure.

In this study, we first explored the cells’ resistance to TRAIL in three bladder cancer cell lines (5637, T24, and BIU 87). The results showed that different bladder cancer cell lines had different sensitivities to TRAIL. 5 ng/mL of TRAIL significantly inhibited the viability of 5637 cells. However, the viabilities of T24 and BIU 87 cells did not change significantly even treated with higher concentrations of TRAIL (20 ng/mL) (Figure 2). Therefore, 5637 was classified as TRAIL-sensitive bladder cancer cells, and T24 and BIU 87 were classified as TRAIL-resistant cancer cells. We also checked the sensitivity of bladder epithelial cell SV-HUC-1 to TRAIL and found SV-HUC-1 was also insensitive to TRAIL.

RH is a natural anthraquinone derivative, which is extracted from the roots of *Polygonum cuspidatum*, *Polygonum multiflorum* or *Rheum rhabarbarum*. Several studies have shown that RH exhibits anti-cancer activities [29, 30]. In our study, we determined the effects of RH on the proliferation of three bladder cancer cell lines. The results showed that RH significantly reduced the survival rate of bladder cancer cells in a dose-dependent manner. However, RH also showed obvious cytotoxicity to SV-HUC-1 as the concentration increased (Figure 3A). Therefore, RH alone may not be suitable for bladder cancer treatment due to its cytotoxicity to normal cells.

Herbs in combination with TRAIL was reported to reverse the sensitivity of TRAIL-resistant tumor cells. For example, Kim et al. [21] had confirmed that Icariin sensitized human colon cancer cells to TRAIL-induced apoptosis via up-regulating DR5 and DR4 expression. Ngai [22] found that Curcumin could up-regulate death receptors of TRAIL (DR4 and DR5) to enhance the apoptosis-inducing effects of TRAIL. Deng et al. [31] found that non-toxic dosage of andrographolide enhanced TRAIL-mediated apoptosis in bladder cancer cells. We decided to test if TRAIL resistance could be reversed by administering non-toxic concentration of RH in bladder cancer cells. First, we determined cell viability and apoptosis after treatment with RH, and 10 μg/mL of RH was demonstrated to be a non-toxic concentration to both bladder cancer cell lines and normal epithelial cells (Figure 3A and 3B). Next, we treated the bladder cancer cell lines and normal epithelial cells with 10 μg/mL of RH in combination with TRAIL. The results showed that this combination significantly inhibited cell growth and enhanced cell apoptosis not only in TRAIL-sensitive bladder cancer cells 5637, but also in both TRAIL-resistant bladder cancer cells T24 and BIU 87. Furthermore, this combination was not toxic to SV-HUC-1 (Figure 4A and 4B). Additionally, as a large number of reports showed that TRAIL-mediated apoptosis is mainly achieved through the caspase-dependent pathways [32]. We detected the expression of apoptosis-related proteins, including caspase 8 and caspase 3 using Western blot. The results showed that the RH and TRAIL combination increased the level of cleaved caspase-8 and cleaved caspase-3 (Figure 4C). These data demonstrated that RH enhanced TRAIL-induced apoptosis in bladder cancer cells.

However, a question remains. Why does non-toxic concentration RH reverse TRAIL resistance? Membrane receptors DR4 and DR5 receive apoptotic signals generated by TRAIL and initiate cell death, and they are considered as regulators of TRAIL-induced apoptosis [33]. We detected the expression levels of DR4 and DR5 after administering the RH combination with TRAIL in the selected cancerous cell lines. We found that RH increased the expression of DR5 protein in bladder cancer cells in a dose-dependent manner, but not DR4 (Figure 5A and 5B). RH also increased DR5 mRNA expression at transcriptional level (Figure 5C). Next, to further evaluate the effects of DR5 in the sensitization of TRAIL-resistant cells by RH, siDR5 was used to knockdown DR5 expression in BIU 87 cells (Supplementary Table 2). Results showed that DR5 mRNA and protein expression (Figure 6A and 6B) were significantly inhibited by siDR5, and TRAIL-induced apoptosis was also effectively inhibited (Figure 6C and 6D). These data suggested that RH enhanced TRAIL-induced apoptosis in bladder cancer cells by up-regulating DR5 expression.

## CONCLUSION

**Figure 7:**
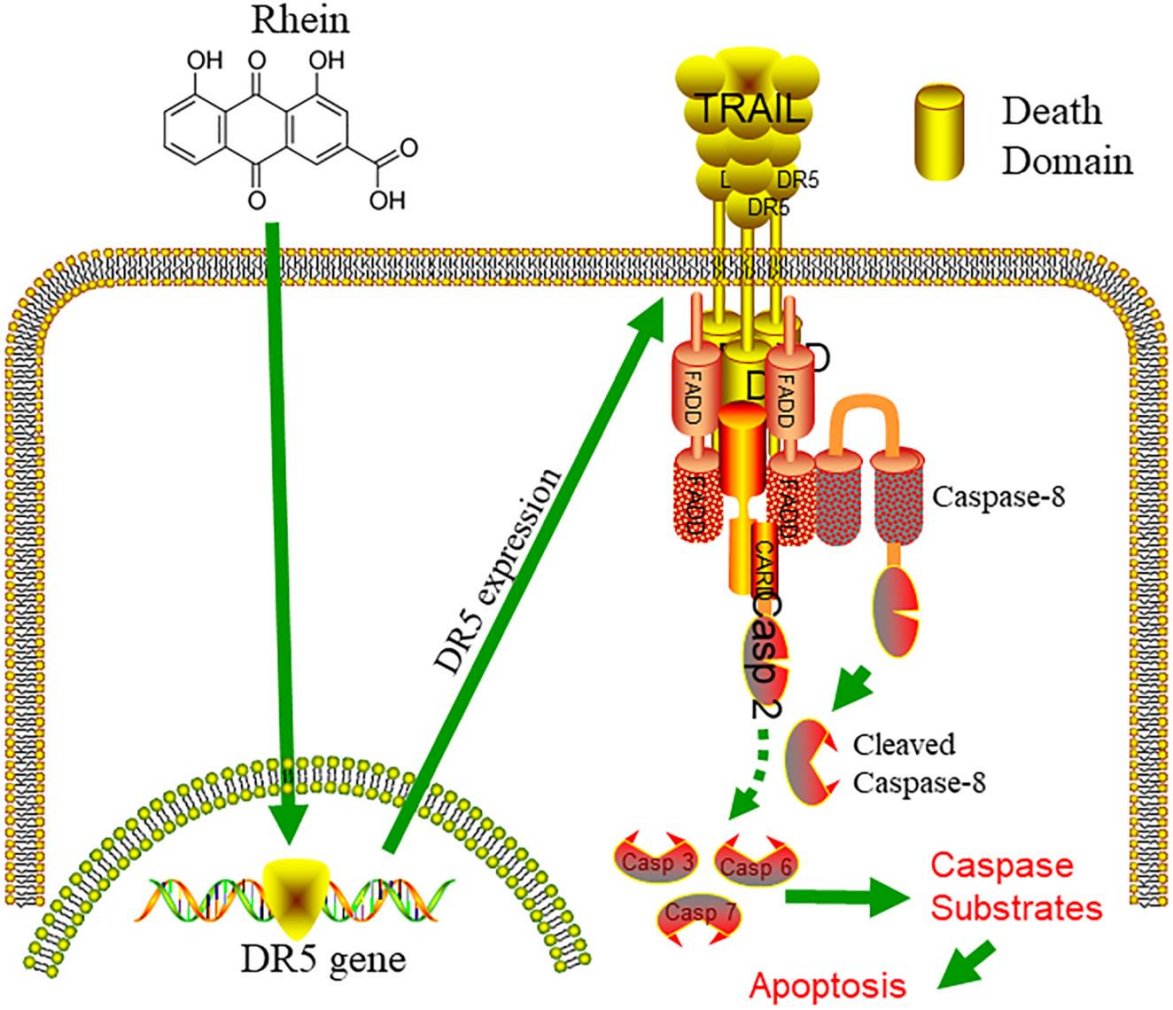
**Graphical abstract.** Combined treatment with TRAIL and non-toxic concentration of RH reversed TRAIL resistance and increased TRAIL-mediated apoptosis in bladder cancer cells by up-regulating DR5 expression.

In this study, we found that non-toxic concentration of RH reversed TRAIL resistance and increased TRAIL-mediated apoptosis in bladder cancer cells by up-regulating DR5 expression (Figure 7). This combination effectively enhanced TRAIL-induced apoptosis in bladder cancer cells and was not cytotoxic to normal bladder epithelial cells, thus provides potential value in the clinical treatment of bladder cancer.

## Supplementary Materials

Supplementary Tables
