# Supplementary material for: Rhein promotes TRAIL-induced apoptosis in bladder cancer cells by up-regulating DR5 expression
Source: Aging (Albany NY). 2022 Aug 19;14(16):6642–55. doi: 10.18632/aging.204236 (PMC9467405; doi:10.18632/aging.204236)
Supplement: Supplementary Tables [file aging-14-204236-s001.pdf]

SUPPLEMENTARY TABLES

Supplementary Table 1. The sequence of primers.

Primer name	Primer sequence	Ref
DR5 1		(Jin et al., 2014)
Forward	5′-CGCTGCACCAGGTGTGATTC-3′	
Reverse	5′-GTCTCCTCCACAGCTGGGAC-3′	
GAPDH		
Forward	5′-ACCCACTCCTCCACCTTTGAC-3′	
Reverse	5′-TGTTGCTGTAGCCAAATTCGTT-3′	

Supplementary Table 2. The sequence of siRNA.

siRNA name	siRNA sequence
siDR5 1	5′-GACCCUUGUGCUCGUUGUCTT-3′
siDR5 2	5′-UCAGAAGACGGUAGAGAUUTT-3′
siDR5 3	5′-GCCUCAUGGACAAUGAGAUTT-3′
NC	5′-UUCUCCGAACGUGUCACGUTT-3′
